# External Validation of a Tool Predicting 7-Year Risk of Developing Cardiovascular Disease, Type 2 Diabetes or Chronic Kidney Disease

**DOI:** 10.1007/s11606-017-4231-7

**Published:** 2017-12-04

**Authors:** Simone P. Rauh, Femke Rutters, Amber A. W. A. van der Heijden, Thomas Luimes, Marjan Alssema, Martijn W. Heymans, Dianna J. Magliano, Jonathan E. Shaw, Joline W. Beulens, Jacqueline M. Dekker

**Affiliations:** 1 Department of Epidemiology and Biostatistics, Amsterdam Public Health Research Institute, VU University Medical Center, Amsterdam, The Netherlands; 2 Department of General Practice and Elderly Care Medicine, Amsterdam Public Health Research Institute, VU Medical Center, Amsterdam, The Netherlands; 3 Unilever Research and Development, Vlaardingen, the Netherlands; 4 Department of Clinical Diabetes and Epidemiology, Baker IDI Heart and Diabetes Institute, Melbourne, Australia; 5 Department of Epidemiology and Preventive Medicine, Monash University, Melbourne, Australia

**Keywords:** prediction tool, generalizability, cardiovascular disease, type 2 diabetes, chronic kidney disease

## Abstract

**Background:**

Chronic cardiometabolic diseases, including cardiovascular disease (CVD), type 2 diabetes (T2D) and chronic kidney disease (CKD), share many modifiable risk factors and can be prevented using combined prevention programs. Valid risk prediction tools are needed to accurately identify individuals at risk.

**Objective:**

We aimed to validate a previously developed non-invasive risk prediction tool for predicting the combined 7-year-risk for chronic cardiometabolic diseases.

**Design:**

The previously developed tool is stratified for sex and contains the predictors age, BMI, waist circumference, use of antihypertensives, smoking, family history of myocardial infarction/stroke, and family history of diabetes. This tool was externally validated, evaluating model performance using area under the receiver operating characteristic curve (AUC)—assessing discrimination—and Hosmer–Lemeshow goodness-of-fit (HL) statistics—assessing calibration. The intercept was recalibrated to improve calibration performance.

**Participants:**

The risk prediction tool was validated in 3544 participants from the Australian Diabetes, Obesity and Lifestyle Study (AusDiab).

**Key Results:**

Discrimination was acceptable, with an AUC of 0.78 (95% CI 0.75–0.81) in men and 0.78 (95% CI 0.74–0.81) in women. Calibration was poor (HL statistic: *p* < 0.001), but improved considerably after intercept recalibration. Examination of individual outcomes showed that in men, AUC was highest for CKD (0.85 [95% CI 0.78–0.91]) and lowest for T2D (0.69 [95% CI 0.65–0.74]). In women, AUC was highest for CVD (0.88 [95% CI 0.83–0.94)]) and lowest for T2D (0.71 [95% CI 0.66–0.75]).

**Conclusions:**

Validation of our previously developed tool showed robust discriminative performance across populations. Model recalibration is recommended to account for different disease rates. Our risk prediction tool can be useful in large-scale prevention programs for identifying those in need of further risk profiling because of their increased risk for chronic cardiometabolic diseases.

## INTRODUCTION

Chronic cardiometabolic diseases, including cardiovascular disease (CVD), type 2 diabetes (T2D) and chronic kidney disease (CKD), are associated with reduced quality of life and are a major cause of death.^1^^–^ 4 As these diseases share many modifiable risk factors, common opportunities for prevention have been suggested.^5^^–^ 7 To improve the cost-effectiveness of prevention programs, a target population is needed,^8^^–^ 10 and valid tools are thus needed to accurately identify individuals at risk for chronic cardiometabolic disease who may benefit most from interventions.

We recently developed such a risk prediction tool, including only non-invasive measures, to predict the combined 7-year risk for CVD, T2D and CKD in the general population.^7^ The tool was developed in a pooled data set of three Dutch cohort studies. It demonstrated the ability to discriminate between those at high and low risk of developing chronic cardiometabolic disease, and it accurately predicted absolute disease risk. The tool is incorporated into the Dutch guidelines for general practitioners, enabling identification of individuals in need of further risk profile assessment (including standard blood tests).^9^^,^ 11 Although the tool demonstrated good internal validation, its performance has not yet been assessed in other populations. As performance is generally overestimated in the development population, external validation of a prediction tool is an essential step in determining its generalizability.^12^^–^ 14

The aim of the current study was therefore to externally validate the previously developed non-invasive risk prediction tool for predicting 7-year risk for chronic cardiometabolic disease, by assessing its discrimination and calibration in the Australian Diabetes, Obesity and Lifestyle Study (AusDiab).

## METHODS

### Original Risk Prediction Tool

The original risk prediction tool was developed in 6780 Caucasian participants (28–85 years) from three Dutch population-based cohorts: the Rotterdam study (1989–1993 and 1997–1999 measurements),^15^ the Hoorn study (1989 and 2000 measurements),^16^ and the Prevention of Renal and Vascular End-Stage Disease (PREVEND) study (1997 and 2005 measurements).^7^^,^ 17 Exclusion criteria were as follows: 1) prevalent diagnosed chronic cardiometabolic disease: 1a) CVD, including myocardial infarction, percutaneous transluminal coronary angioplasty, coronary artery bypass graft, angina pectoris, stroke, intermittent claudication, peripheral arterial intervention, or heart failure; 1b) T2D defined by self-reported T2D and/or use of antidiabetic medication; or 1c) CKD defined by self-reported or estimated glomerular filtration rate (eGFR) < 15 mL/min/1.73 m^2^; 2) no follow-up information on the three diseases of interest; 3) death of other than cardiovascular causes during follow-up.

The tool was stratified for sex and included age, BMI, waist circumference, use of antihypertensives, smoking, family history (parent and/or sibling) of myocardial infarction or stroke (< 65 years), and family history (parent and/or sibling) of diabetes (Table 1). The outcome was incident chronic cardiometabolic disease, defined as follows: nonfatal CVD as described above, fatal CVD defined by ICD-10 codes I00–I99, sudden death (ICD-10 code R96), T2D defined by fasting plasma glucose, post-load plasma glucose, or by use of antidiabetic medication, and/or CKD defined by eGFR <60 mL/min/1.73 m^2.^ 7 Internal validation was performed using bootstrapping techniques,^7^ resulting in a calibration slope of 0.97 for men and 0.98 for women.

**Table 1:** Original Risk Prediction Tools for Men and Women (Logistic Regression Models^7^)

	Men		Women	
	Regression coefficient	OR (95% CI)	Regression coefficient	OR (95% CI)
Age (years)				
< 45	[Reference]			
45–49.9	0.91	2.5 (1.2–5.0)	0.69	2.0 (1.0–4.1)
50–54.9	1.20	3.3 (1.7–6.4)	1.08	2.9 (1.6–5.5)
55–59.9	1.57	4.8 (2.7–8.7)	1.54	4.7 (2.6–8.2)
60–64.9	2.34	10.4 (5.8–18.6)	1.98	7.2 (4.1–12.7)
65–69.9	2.66	14.3 (7.9–25.7)	2.55	12.8 (7.3–22.5)
70–74.9	3.26	25.9 (14.2–47.5)	3.34	28.1 (15.8–50.1)
75–84.9	4.29	72.8 (37.6–140.9)	4.06	58.2 (31.9–106.1)
BMI (kg/m^2^)				
< 25	[Reference]			
25–29.9	0.32	1.4 (1.1–1.7)	0.27	1.3 (1.1–1.6)
≥ 30	0.87	2.4 (1.6–3.6)	0.52	1.7 (1.3–2.2)
Waist (cm)				
Men <94; women <80	[Reference]			
Men 94–101.9; women 80–87.9	0.20	1.2 (1.0–1.5)	0.12	1.1 (0.9–1.4)
Men >102; women >88	0.19	1.2 (0.9–1.6)	0.40	1.5 (1.2–1.9)
Use of antihypertensives	0.74	2.1 (1.6–2.7)	0.75	2.1 (1.8–2.6)
Current smoking	0.63	1.9 (1.5–2.3)	0.61	1.8 (1.5–2.2)
Parent and/or sibling with MI or stroke (age < 65)	0.09	1.1 (0.9–1.4)	0.26	1.3 (1.1–1.5)
Parent and/or sibling with diabetes	0.30	1.3 (1.1–1.7)	0.21	1.2 (1.0–1.5)

BMI, body mass index; MI, myocardial infarction

Calibration slope: 0.97 for men and 0.98 for women. Intercept after internal validation: −3.497 for men and −3.793 for women

### External Validation Data Set

The design and methodology of the AusDiab study have been described previously.^18^ In short, AusDiab is a population-based survey of 11,247 Europid adults aged ≥25 years, recruited in 1999–2000.^18^^,^ 19 A total of 6400 and 4614 participants returned for a 5-year follow-up (2004–2005) and a 12-year follow-up (2012), respectively.^20^ All participants consented to participate.

To externally validate the previously developed tool over a similar follow-up period, we used the data for 6400 participants from 2004–2005 as baseline, and we used the 2012 measurement as follow-up. In line with the development data, we excluded participants as follows: 1) age ≥ 85 years (*n* = 73); 2) previous diagnosis of chronic cardiometabolic disease (*n* = 482): 2a) prevalent CVD, including myocardial infarction, percutaneous transluminal coronary angioplasty, coronary artery bypass graft or stroke; 2b) prevalent known T2D defined by self-report confirmed with glucose measurements and/or in combination with self-reported use of antidiabetic medication, or 2c) prevalent CKD defined by eGFR <15 mL/min/1.73 m^2^; 3) no information on chronic cardiometabolic disease at baseline (*n* = 338); 4) died during follow-up from other than cardiovascular causes (*n* = 175, including three with ICD-10 code R99 [unknown cause of death] and four for whom cause could not be retrieved); or 5) no follow-up data on chronic cardiometabolic disease (*n* = 1635, including 768 who did not attend the 2012 follow-up measurement and 583 who only attended the 2012 phone questionnaire). Participants with undiagnosed T2D or CKD at baseline (by elevated glucose measurements or based on eGFR levels 15–60 mL/min/1.73 m^2^) were considered new T2D and CKD cases and were thus not excluded at baseline. This is in line with the development data and with application of the tool in practice, where a portion of diseased individuals will also be undiagnosed. In addition, we excluded 153 participants (4.1%) because information about one of the predictors was missing, resulting in 3544 participants eligible for analysis.

### Outcome and Predictors in External Validation Set

The outcome, developing chronic cardiometabolic disease during follow-up, was defined in line with the development data as much as possible as nonfatal or fatal CVD—including sudden death—and/or T2D and/or CKD. T2D was diagnosed according to the 1999 World Health Organization (WHO) criteria.^21^^,^ 22 CKD was defined as an eGFR <60 ml/min/1.73m^2.^ 23 The cause of death was known through 30 November 2011. Deaths after this date could not be defined as cardiovascular, and these cases were thus excluded. Fatal CVD was determined using ICD-10 codes I00-I99, and sudden death by ICD-10 code R96. Nonfatal CVD was determined by adjudicating self-reported events through 11 April 2011 using medical records,^24^ and was defined as having one of the diseases as described above. Information about angina pectoris, intermittent claudication, peripheral arterial intervention and heart failure was not available in the AusDiab study.

All predictors were defined in line with the development data, except for the predictor ‘family history of myocardial infarction or stroke’; this predictor was not available in the AusDiab study and was therefore excluded. As an additional analysis, we evaluated the performance of the tool without this predictor in the development data.

### Statistical Analysis

For each AusDiab participant, the probability of developing chronic cardiometabolic disease was calculated by applying the regression coefficients of the original logistic regression models for men and women, including the calibration slope and the intercept after internal validation, in the development study. Next, the performance of the tools for men and women was assessed in terms of discrimination (the tool’s ability to distinguish between those at high and low risk for developing chronic cardiometabolic disease) and calibration (the tool’s ability to accurately predict absolute disease risk). For discrimination, the area under the receiver operating characteristic curve (AUC) was assessed, considering AUCs of 0.70–0.79 and ≥0.80 as indicating acceptable and excellent discrimination, respectively.^25^ Calibration was assessed by the Hosmer–Lemeshow goodness-of-fit (HL) statistic (non-significant values indicating adequate calibration) and by calibration graphs plotting predicted chronic cardiometabolic disease risk against the observed rates in deciles of predicted risk. Ideally, these predicted risk values would be on the 45-degree line, indicating that rates of predicted risk values equal the observed rates throughout the entire risk spectrum. In addition, we calculated observed-to-expected (O/E) ratios by dividing observed disease rates by predicted disease risk.^26^ Ratios below 1.0 indicate overestimation of risk, while ratios above 1.0 indicate underestimation.

Differences in disease rates between the two data sets led to a significant deviation between predicted and observed disease risk in the AusDiab study. Therefore, the tool was recalibrated to improve performance in the AusDiab study by adjusting the intercept of the model: the regression coefficients of the original tool were applied to the AusDiab study and fixed at their original values, while a new intercept was estimated as the only free parameter.^27^ After recalibration of the intercept, calibration was again assessed. The added value of the newly estimated intercept was tested for significance using the Wald statistic, considering a two-sided *p*-value of <0.05 as statistically significant.

As an additional analysis, the discriminative performance of our tool for the individual outcome CVD was compared to the performance of the non-invasive CVD risk score described by Gaziano et al.^28^ in the present data set. This risk score is stratified by sex and includes the predictors age, systolic blood pressure, current smoking, BMI, history of blood pressure treatment, and history of diabetes. Furthermore, discriminative performance of our tool for the individual outcome T2D was compared to the performance of the Finnish diabetes risk score^29^ in the present data set. This risk score includes the predictors age, BMI, waist circumference, use of antihypertensive medication, and history of high blood glucose. The performance of our tool for the individual outcome CKD could not be compared to the performance of a CKD-specific risk score in the present data set, since to our knowledge, all non-invasive scores predicting CKD incidence include prevalent T2D and/or CVD as important predictors.^30^^,^ 31

Statistical analyses were performed using SPSS version 22 (IBM Corp., Armonk, NY, USA) and R software version 3.2.5, using the packages ‘rms’.

## RESULTS

### Participant Characteristics

The incidence of chronic cardiometabolic disease was 15% for men and 12% for women over a mean follow-up period of 6.9 (SD 0.3; IQR: 6.7–7.2) years in the AusDiab study, compared to 36% for men and 34% for women over mean follow-up of 6.9 (SD 1.1; IQR: 6.3–7.0) years in the development data^7^ (Table 2). The incidence of CVD, T2D and CKD was 4%, 6% and 4% in the AusDiab study, respectively, compared to 16%, 12% and 13% in the development data.^7^

**Table 2:** Baseline Characteristics and Incidence of the Outcome in the AusDiab Study

	Men	Women
Baseline characteristics		
No. (%)	1563 (44.1%)	1981 (55.9%)
Age (years)	54.4 ± 11.3	53.9 ± 11.0
< 45	304 (19.4%)	407 (20.5%)
45–49.9	231 (14.8%)	333 (16.8%)
50–54.9	282 (18.0%)	323 (16.3%)
55–59.9	273 (17.5%)	316 (16.0%)
60–64.9	170 (10.9%)	263 (13.3%)
65–69.9	125 (8.0%)	170 (8.6%)
70–74.9	97 (6.2%)	94 (4.7%)
75–84.9	81 (5.2%)	75 (3.8%)
BMI (kg/m^2^)	27.7 ± 4.1	27.1 ± 5.4
< 25	418 (26.7%)	801 (40.4%)
25–29.9	775 (49.6%)	693 (35.0%)
> 30	370 (23.7%)	487 (24.6%)
Waist (cm)	98.1 ± 11.4	86.0 ± 12.6
Men <94; women <80	577 (36.9%)	694 (35.0%)
Men 94–101.9; women 80–87.9	472 (30.2%)	502 (25.3%)
Men >102; women >88	514 (32.9%)	785 (39.6%)
Use of antihypertensives	266 (17.0%)	351 (17.7%)
Current smoking	160 (10.2%)	130 (6.6%)
Parent and/or sibling with diabetes	367 (23.5%)	544 (27.5%)
Incidence of the outcome		
Cardiometabolic disease (composite outcome)	241 / 1563 (15.4%)	232 / 1981 (11.7%)
Cardiovascular disease	87 / 1563 (5.6%)	40 / 1981 (2.0%)
Type 2 diabetes	119 / 1513 (7.9%)	104 / 1949 (5.3%)
Chronic kidney disease	46 / 1499 (3.1%)	104 / 1949 (5.3%)

BMI, body mass index

Values are mean ± SD or number (%)

### Performance of the Risk Prediction Tool

The tool showed acceptable discrimination in the AusDiab study, with AUC of 0.78 (95% CI 0.75–0.81) in men and 0.78 (95% CI 0.74–0.81) in women (Table 3), compared to 0.80 (95% CI 0.78–0.82) and 0.82 (95% CI 0.81–0.83), respectively, in the development data.

**Table 3:** Model Performance in the AusDiab Study

		Original risk prediction tool after internal validation applied to AusDiab study		Model with adjusted intercept	
		Men	Women	Men	Women
AUC (95% CI)	Chronic cardiometabolic disease	0.78 (0.75–0.81)	0.78 (0.74–0.81)	0.78 (0.75–0.81)	0.78 (0.74–0.81)
	Cardiovascular disease	0.82 (0.77–0.86)	0.88 (0.83–0.94)	0.82 (0.77–0.86)	0.88 (0.83–0.94)
	Type 2 diabetes	0.69 (0.65–0.74)	0.71 (0.66–0.75)	0.69 (0.65–0.74)	0.71 (0.66–0.75)
	Chronic kidney disease	0.85 (0.78–0.91)	0.79 (0.74–0.83)	0.85 (0.78–0.91)	0.79 (0.74–0.83)
HL statistic, χ^2^ (*p*-value)*		158.67 (*p* < 0.001)	115.74 (*p* < 0.001)	16.18 (*p* = 0.040)	32.14 (*p* < 0.001)
O/E ratio		0.58	0.62	1.00	1.02
Model parameters	Total intercept	−3.50	−3.79	−4.40	−4.52
	Calibration intercept (*p*-value)^†^	–	–	−0.90 (*p* < 0.001)	−0.73 (*p* < 0.001)

AUC, area under the receiver operating characteristic curve; HL statistic, Hosmer–Lemeshow goodness-of-fit statistic; O/E ratio, observed-to-expected ratio; χ^2^, chi-square

*HL statistic: non-significant *p*-values indicate adequate fit

^†^Deviation from original intercept after internal validation

**Figure 1:**
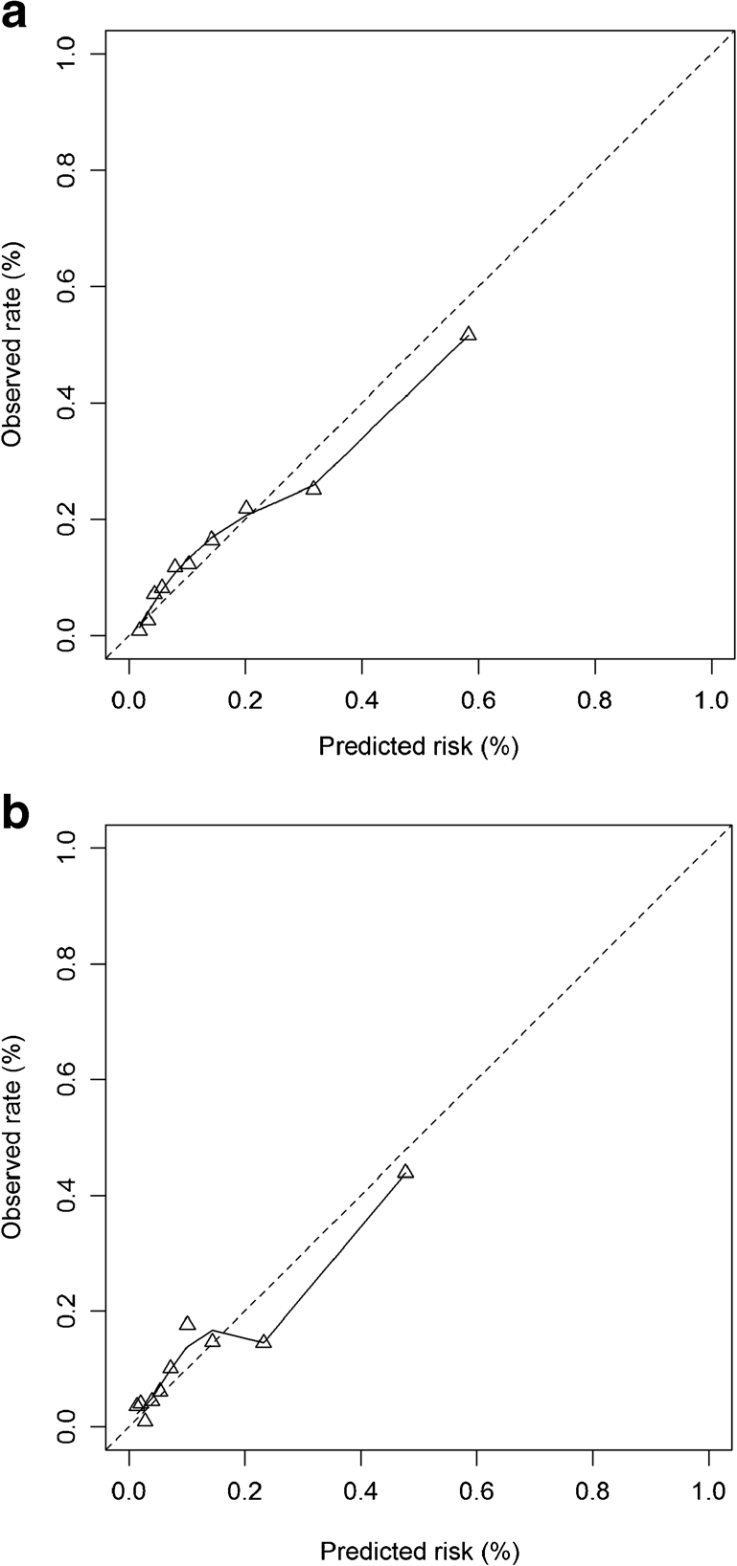
Calibration plots of the risk prediction tool (after internal validation) with recalibrated intercept for men (**a**) and women (**b**). The dotted line indicates perfect calibration. The triangles represent the observed and expected mortality rates in deciles of predicted mortality risk. The solid line is a smoothed spline curve.

As expected based on the lower disease incidence in the AusDiab study, calibration was initially poor for men and women (HL statistic: *p* < 0.001), systematically overestimating disease risk (Table 3). Adjusting the model intercept for the AusDiab study improved calibration: although the HL statistic was still significant, the calibration graphs showed adequate calibration for men (Fig. 1a) and women (Fig. 1b).

Additionally, in men we observed that the AUC was highest for CKD (0.85 [95% CI 0.78–0.91]), followed by CVD (0.82 [95% CI 0.77–0.86]), and lowest for T2D (0.69 [95% CI 0.65–0.74]). In women, the AUC was highest for CVD (0.88 [95% CI 0.83–0.94]), followed by CKD (0.79 [95% CI 0.74–0.83]), and lowest for T2D (0.71 [95% CI 0.66–0.75]). The discriminative performance for the individual diseases was generally comparable to that for the development data.^7^

Applying the tool without the predictor ‘family history of myocardial infarction or stroke’ to the development data led to a decrease in AUC from 0.82 to 0.79 (95% CI 0.78–0.81) in women, while AUC did not change for men.

The performance of the Gaziano et al.^28^ tool in the present data was comparable to the performance of our tool for predicting CVD, with an AUC of 0.82 (95% CI 0.78–0.86) for men and 0.87 (95% CI 0.82–0.93) for women. The performance of the Finnish diabetes tool^29^ was better than that of our tool for predicting T2D, with an AUC of 0.75 (95% CI 0.71–0.80) for men and 0.76 (95% CI 0.72–0.80) for women.

## DISCUSSION

The aim of this study was to externally validate a previously developed risk prediction tool predicting 7-year risk for chronic cardiometabolic diseases.^7^ In an Australian population-based cohort with lower disease incidence than in the development data, the tool performed well with regard to discrimination. Calibration was poor, systematically overestimating disease rates, but this improved substantially by recalibration of the tool to account for differences in disease rates. When studied separately for the individual diseases, in men the tool discriminated best for CKD and worst for T2D. In women, the tool discriminated best for CVD and worst for T2D.

Compared to the development data, there was only a small decrease in discriminative performance in this external validation study, indicating that the tool can be used without adaptation for discriminating between low-risk and high-risk individuals in different populations. Further, this may indicate that overestimation of model performance, which is often reported when assessing performance in the development data,^12^ was scarcely present in the development study. This could be due to the internal validation procedure that was part of tool development. In addition, the tool was developed in three slightly different populations, which may have contributed to robust performance across populations.

In predicting individual diseases, we found that the Finnish diabetes risk score had better discriminative performance than our tool for the prediction of T2D only in this data set. On the other hand, for the prediction of CVD only, the discriminative performance of our combined risk prediction tool was comparable to that of the Gaziano et al. non-invasive CVD tool in this data set.^28^ Although a disease-specific tool might be more accurate for the prediction of T2D only, it ignores the risk for the other two diseases, and the added value of a tool predicting the combination of three chronic metabolic diseases may still outweigh the small reduction in discrimination for T2D specifically. For prevention programs, our combined risk prediction tool provides a unique opportunity for multiple risk factor screening and treatment. This might be more relevant for disease prevention strategies than simply focusing on one of these diseases, as well as more user friendly and less confusing, enabling the use of one tool instead of three with possibly different outcomes.

The initially poor calibration in the AusDiab study can be explained by the lower chronic cardiometabolic disease incidence than in the development data: 13% vs. 35%. This lower incidence might be due to several factors. First, the definition of CVD in the AusDiab study differed from the definition in the development data, since no information was available on angina pectoris, peripheral arterial intervention, intermittent claudication or heart failure. Second, there were differences in population characteristics, including a lower age and lower smoking prevalence. Third, this data set had a shorter follow-up duration for nonfatal CVD. Finally, the AusDiab study was conducted during a different time period, 2004–2012, versus 1989–2005 for the development cohorts; the increased use of antihypertensives and statins in this later time period^32^^,^ 33 may have influenced performance of the model in the AusDiab cohort.

Adjusting the intercept for the AusDiab study improved calibration considerably, in line with previous research indicating that simple recalibration techniques seem sufficient for improving performance, especially when discrimination is already adequate in a new setting.^27^^,^ 34 ^,^ 35 This indicates that the discriminative performance of our tool may be robust for different settings, while calibration may be inadequate in settings with different chronic cardiometabolic disease rates.

In line with the development data, we excluded patients with known T2D and CKD at baseline, but those identified by screening at baseline were considered as cases. The rationale behind this was that individuals with undiagnosed T2D or CKD cannot be excluded from using the tool, but should rather be identified.^7^ Exclusion of prevalent undiagnosed cases led to only a minor decrease in discriminative performance of the tool in the development data.^7^

There are some limitations of this study that should be discussed. First, the tool has been developed and validated in predominantly Europid populations and might not be transferable to different populations. Therefore, the tool’s performance should be evaluated before applying it to other ethnic groups. Second, the AusDiab study lacked data on family history of myocardial infarction or stroke. This predictor was therefore excluded. In the development data, applying the tool without this predictor led to a decrease in AUC for women but not for men. Excluding this predictor in the AusDiab study thus may have also led to an underestimation of the AUC for women. Third, in the AusDiab study and in the development studies, previous non-response analyses showed selective participation in follow-up of participants who were relatively healthy at baseline^7^^,^ 20; this may have led to an underestimation of disease rates and would underestimate risk when the model is applied in clinical practice. Therefore, we recommend model recalibration in settings where different rates are expected. Finally, as mentioned above, CVD incidence was somewhat underestimated in the AusDiab study. A higher CVD incidence might have resulted in better calibration performance. However, despite these differences in outcome measurement, the discriminative performance of the tool was good, and updating the intercept improved calibration performance. In prevention programs, the tool can therefore be used in settings where 7-year chronic cardiometabolic disease rates, as defined in accordance with the development data, are expected to be comparable to the rate in the development data. As noted above, we recommend model recalibration in settings where different rates are expected.

In the Netherlands, this risk prediction tool is freely available for patients via health organization websites, referring individuals at highest risk to their general practitioner. In addition, the tool is incorporated into the Dutch guidelines for general practitioners, ‘The Prevention Visit’.^9^ These guidelines describe the screening for chronic cardiometabolic diseases as a stepwise approach. Our tool serves as the first step in screening, in order to differentiate between people in need of further risk assessment and those at low risk.^9^^,^ 11 Assessing the external validity of the tool in another population was an important step, and the present study results add strength to the validity of the tool.^12^^–^ 14 The effectiveness and cost-effectiveness of using this tool in combination with a tailored lifestyle intervention are currently being studied.^36^^,^ 37

In conclusion, our non-invasive risk prediction tool predicting 7-year-risk for chronic cardiometabolic disease showed good generalizability regarding discriminative performance. Recalibration of the tool is recommended before applying it to settings where different chronic cardiometabolic disease rates are expected. Our risk prediction tool can be useful in prevention programs as a first step in the identification of individuals who are in need of further multifactorial risk assessment and possible intervention given their increased risk for CVD, T2D or CKD.
